# Comparison of Radiographic Outcomes Between TPLO + TTT and CTWO + TTT in Dogs with Concurrent Cranial Cruciate Ligament Rupture and Medial Patellar Luxation

**DOI:** 10.3390/ani16182912

**Published:** 2026-09-16

**Authors:** Hyun-Joon Roh, Jeonghyun Seo, Chun-Sik Bae

**Affiliations:** 1 Department of Veterinary Surgery, College of Veterinary Medicine, Chonnam National University, Gwangju 61186, Republic of Korea; 2 KINTEX Coolpet Animal Hospital, 171 Kintex-ro, Ilsanseo-gu, Goyang-si 10390, Republic of Korea; 3 Time Animal Medical Center, 57 Dunsan-ro, Seo-gu, Daejeon 35233, Republic of Korea

**Keywords:** TPLO, CTWO, tibial tuberosity transposition, cranial cruciate ligament rupture, medial patellar luxation, normalized effective tibial length, Guénégo–Verwaerde index, patella baja, radiographic assessment

## Abstract

Cranial cruciate ligament (CrCL) rupture frequently occurs with medial patellar luxation (MPL) in small-breed dogs, and both conditions may be corrected during surgery using a proximal tibial osteotomy—tibial plateau leveling osteotomy (TPLO) or cranial tibial wedge osteotomy (CTWO)—combined with tibial tuberosity transposition (TTT). Because these osteotomies differ in patellar ligament displacement, they may interact differently with TTT. We retrospectively compared TPLO + TTT and CTWO + TTT in 20 stifles from 18 small-breed dogs. Tibial length was quantified using a normalized effective tibial length (NEL) referenced to the tibial mechanical axis, and patellar position was assessed using the Guénégo–Verwaerde index (GVI). Both outcomes were expressed as the within-limb relative changes to reduce the influence of positioning differences. NEL changed by −5.65 ± 1.29% after CTWO + TTT and −0.98 ± 0.93% after TPLO + TTT, with non-overlapping ranges in this sample. Postoperative GVI decreased in all 10 CTWO + TTT stifles (mean −14.6%) but was unchanged after TPLO + TTT (+1.7%, *p* = 0.73; between-group *p* = 0.002). Patellar descent corresponded to only about 26% of tibial shortening. Radiographic union was more advanced after TPLO + TTT at 8–10 weeks, although callus was present in every stifle and no non-union occurred. TPLO + TTT preserved tibial length and the patella–tibial plateau relationship.

## Simple Summary

Cranial cruciate ligament rupture often occurs together with medial patellar luxation in small-breed dogs, and both are usually corrected during the same surgery. The cruciate problem is treated by reshaping the top of the shin bone—either by rotating a curved cut (tibial plateau leveling osteotomy, TPLO) or by removing a wedge of bone (cranial tibial wedge osteotomy, CTWO)—while the luxating kneecap is treated by moving the bony attachment of the kneecap tendon sideways (tibial tuberosity transposition, TTT). Because the wedge technique removes bone, it shortens the shin bone and pulls the kneecap downward, so the surgeon must also move the attachment upward to compensate—a correction limited by the bone available. We reviewed the radiographs of small-breed dogs treated with either technique combined with TTT. The wedge technique shortened the bone and lowered the kneecap in every limb, whereas the rotational technique preserved both. Healing of the bone cut was further advanced with the rotational technique at the time of assessment, although bone was uniting in all dogs. These findings suggest that when the kneecap position must also be corrected, the rotational technique places a smaller corrective burden on the surgeon.

## 1. Introduction

Cranial cruciate ligament (CrCL) rupture is one of the most common orthopedic conditions in dogs [1,2] and frequently occurs concurrently with medial patellar luxation (MPL) in small-breed dogs [3,4,5,6,7,8]. When these lesions coexist, both are usually corrected during a single surgical procedure. In this setting, cruciate lesion is the primary source of instability and is treated with a proximal tibial osteotomy, whereas MPL is addressed by adding tibial tuberosity transposition (TTT). The relevant clinical question is therefore not which osteotomy is preferable in isolation, but which is more suitable for combination with an additional patellar procedure.

Among the surgical options for CrCL rupture, tibial plateau leveling osteotomy (TPLO) has become a standard technique that reduces the tibial plateau angle (TPA) to neutralize cranial tibial thrust [9,10]. Cranial tibial wedge osteotomy (CTWO) achieves the same biomechanical goal as TPA reduction by different means and is preferred by some surgeons [11,12]. Surgical correction of MPL primarily involves TTT to improve patellar tracking [13,14].

The two osteotomies interact differently with TTT because of their geometry. TPLO rotates the proximal fragment about a radial cut and does not displace the origin of the patellar ligament, so that tibial length and the relationship between the patella and the tibial plateau are largely preserved; lateral transposition of the tuberosity is therefore generally sufficient for MPL correction. CTWO, in contrast, removes a cranially based wedge, which shortens the tibia in proportion to the wedge angle [12] and displaces the tibial tuberosity—and with it the origin of the patellar ligament—distally [15]. To prevent the patella from descending, the tuberosity must therefore be moved laterally and proximally during TTT, a correction that is constrained by the available proximal bone stock. An ex vivo comparison in the same dogs has confirmed that the wedge procedure exerts substantially greater downward traction on the patella than TPLO [15], and a clinical study using the Guénégo–Verwaerde index (GVI) reported a comparable reduction in patellar height after a modified closing wedge osteotomy [16].

These consequences may be particularly relevant in small-breed dogs, in whom CrCL rupture and MPL most often coexist and in whom a given absolute shortening represents a larger proportion of tibial length. Nevertheless, clinical studies directly comparing the two osteotomies in this population are scarce, and quantitative data on tibial shortening and the resulting patellar position are particularly limited. Conventional effective tibial length (ETL) measurements are also vulnerable to differences in radiographic positioning, which are difficult to standardize in a retrospective clinical series. We therefore investigated how the choice of proximal tibial osteotomy affects the outcome of concurrent TTT in small-breed dogs with CrCL rupture and MPL. Specifically, we aimed to (1) introduce a normalized effective tibial length (NEL), referenced to the tibial mechanical axis, so that the geometric consequences of each osteotomy can be quantified with reduced sensitivity to radiographic positioning; (2) apply the GVI, expressed as a relative change from the preoperative value, to assess the resulting patellar position; and (3) compare the radiographic outcomes of CTWO + TTT and TPLO + TTT, including bone healing. To our knowledge, this normalization has not previously been described.

## 2. Materials and Methods

### 2.1. Study Design and Animals

This retrospective comparative study was conducted at a single institution where more than 400 stifles underwent surgical stabilization for CrCL rupture between May 2020 and December 2025. From this population, cases were identified for the present analysis according to the following criteria: (1) concurrent CrCL rupture and medial patellar luxation (MPL) corrected in a single surgical session by TPLO + TTT or CTWO + TTT, performed by one surgeon; and (2) adequate preoperative, immediate postoperative, and follow-up radiographs were available at the planned time points. Cases were excluded if the required radiographic series was incomplete, where radiographic positioning deviated substantially from a true lateral projection, where implant failure required revision that precluded assessment of union at the planned time points, or where the patellar joint-surface length (PJSL) differed by more than ±8% between time points. Twenty stifles from 18 dogs met all criteria and were analyzed (CTWO + TTT, 10 stifles; TPLO + TTT, 10 stifles). For the two dogs that underwent bilateral surgery, each limb was treated as an independent unit of analysis. The CTWO + TTT procedures were performed earlier in the study period than the TPLO + TTT procedures, because the surgeon had changed technique during the series.

### 2.2. Surgical Procedures

**Figure 1:**
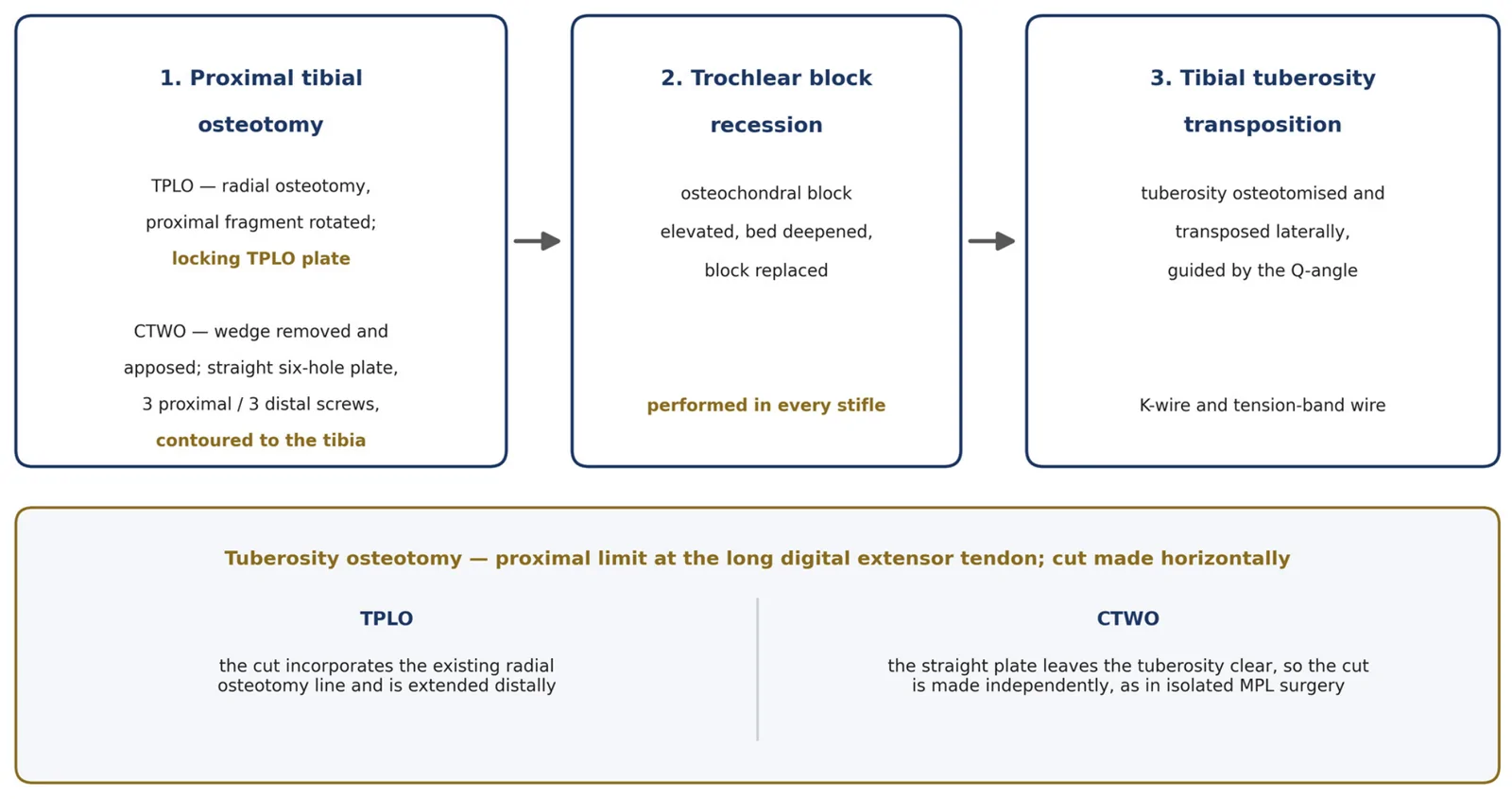
Sequence of the surgical procedure. The proximal tibial osteotomy (TPLO or CTWO) was performed and stabilized first, followed by trochlear block recession, and finally tibial tuberosity transposition. TPLO was stabilized with a locking TPLO plate; CTWO was stabilized with a straight six-hole locking plate contoured to the cranial tibial surface, with three screws proximal and three distal to the osteotomy. Trochlear block recession was performed in every stifle in both groups. The proximal limit of the tuberosity osteotomy was the long digital extensor tendon, from which the cut was made horizontally before lateral transposition. In the TPLO + TTT group, the tuberosity cut incorporated the existing radial osteotomy line and was extended distally from it; in the CTWO + TTT group, the straight plate left the tuberosity unobstructed, so that the cut could be made independently, as in isolated correction of medial patellar luxation.

All surgeries were performed by a single surgeon. TPLO was performed according to the standard technique, with the proximal fragment rotated to a preplanned tibial plateau angle (TPA) and stabilized with a locking TPLO plate (DePuy Synthes, West Chester, PA, USA). CTWO was performed according to the standard technique with a cranially based wedge osteotomy of the proximal tibia. In this group, fixation was achieved with a straight six-hole locking plate (Biortho Medical Science and Technology Co., Ltd., Zhangjiagang, Jiangsu, China) rather than a TPLO plate, using three screws proximal and three screws distal to the osteotomy; the plate was contoured to the cranial tibial surface before application so that it conformed to the shape of the bone. A straight plate was selected because it leaves the tibial tuberosity unobstructed, so that the tuberosity could subsequently be osteotomised independently. In every stifle in both groups, a trochlear block recession was then performed to deepen the femoral trochlear groove. Tibial tuberosity transposition was performed last. The proximal limit of the tuberosity osteotomy was the long digital extensor tendon, from which the cut was made horizontally; in the TPLO + TTT group the cut incorporated the existing radial osteotomy line and was extended distally from it, whereas in the CTWO + TTT group it was made independently, as in isolated correction of medial patellar luxation. The tuberosity was transposed laterally, with the extent of transposition determined by the quadriceps (Q) angle so that the patella tracked in alignment with the lateral trochlear ridge, and caudalization was performed additionally when indicated by the patellar position. In the CTWO + TTT group, the tuberosity was also proximalized with the intention of offsetting the height of the resected wedge; because the achievable extent of proximalization is constrained by the available proximal bone stock, a fixed quantitative target in millimeters was not applied, and the achieved proximalization was not recorded as a separate measurement. The tuberosity was fixed with a Kirschner pin and tension-band wire (Top Medical Company, Seoul, Republic of Korea). The operative sequence is summarized in Figure 1.

### 2.3. Radiographic Measurements

#### 2.3.1. Timing and Positioning

The mediolateral projection used in this study was acquired primarily as a tibial compression stress view for the assessment of cranial cruciate ligament insufficiency, with the tibia held at approximately 90° to the cassette and the femur angled accordingly. This reflects the clinical priority of the present cohort: CrCL rupture was the primary lesion requiring correction, with MPL treated concurrently; therefore, diagnostic assessment of cruciate stability took precedence in the imaging protocol. A true lateral projection was defined by superimposition of the femoral condyles. On remeasurement of all 20 stifles, the resulting stifle joint angle ranged from approximately 110° to 140°, with a comparable distribution in the two groups. Radiographs were obtained preoperatively, immediately postoperatively, and at follow-up; follow-up examinations were performed at 3–4 weeks and at 8–10 weeks postoperatively, and all follow-up outcome measures reported here (GVI and bone healing score) were those assessed at the 8–10-week examination. For each stifle, all time points were radiographed using the same technique, both for consistency of measurement and to allow assessment of bone union and implant position. Radiographs were acquired using a high-frequency X-ray generator (SM-120HFH with an XD9-4 tube; IRETECH, Daejeon, Republic of Korea). All measurements were performed by a single observer (the author) using a picture archiving and communication system (VXV Console, version 2.0.4; Rayence Co., Ltd., Hwaseong-si, Gyeonggi-do, Republic of Korea). We acknowledge that this positioning exceeds the 70–110° range over which patellar height indices have been reported to be stable, and that patellar height measured radiographically varies with stifle flexion; absolute GVI and NEL values were therefore not compared between groups, and all between-group comparisons were based on the within-limb relative change from the preoperative value, which largely mitigates, but does not entirely remove, the influence of positioning.

#### 2.3.2. Tibial Landmark Definitions (Cranial-Cortex Reference)

All tibial landmarks were referenced to the cranial cortex to standardize measurements and reflect the anatomical differences between the two procedures. Intercondylar eminence (IE): most proximal point of the tibial intercondylar eminence. Distal articular center (DA): center of the distal articular surface of the tibia. Osteotomy line (Ost): in CTWO, the point at which the cranial cut surface of the wedge osteotomy meets the cranial cortex; in TPLO, the cranial endpoint at which the radial osteotomy line meets the cranial cortex.

#### 2.3.3. Normalized Effective Tibial Length (NEL)

**Figure 2:**
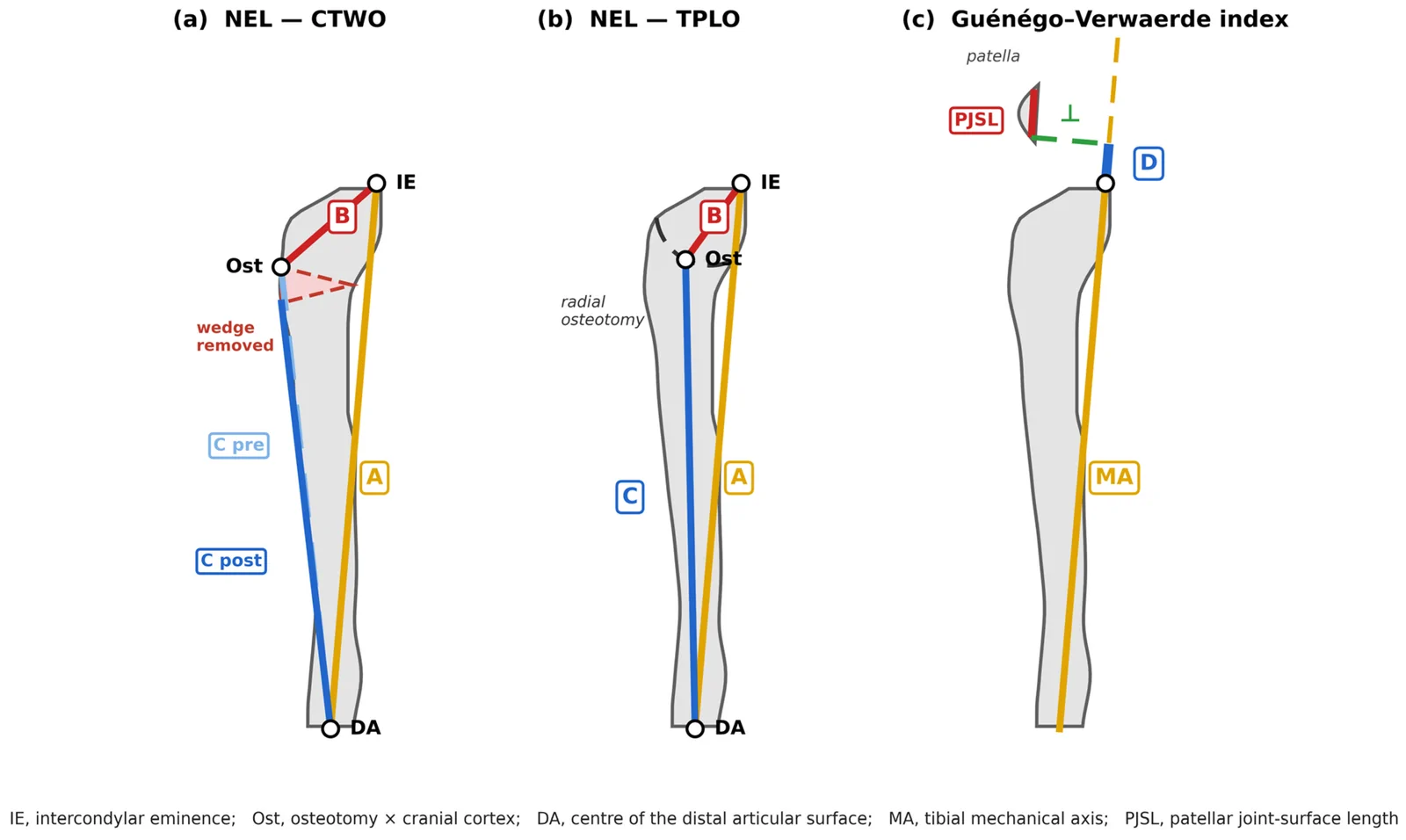
Schematic illustration of the measurements used in this study; cranial is to the left in each panel. (**a**) Normalized effective tibial length (NEL) for CTWO. IE, intercondylar eminence (the most proximal point of the tibia); Ost, the point at which the osteotomy meets the cranial cortex; DA, center of the distal articular surface. A (yellow) is the straight IE → DA distance representing the tibial mechanical axis, B (red) is the IE → Ost proximal segment, and C (blue) is the Ost → DA distal segment. Because a cranially based wedge is removed and the cut surfaces are apposed, B is unchanged, whereas C shortens: the pre-operative C (pale blue, dashed) runs from Ost to DA, and the post-operative C (blue) runs from the distal cranial corner of the resected wedge to DA. (**b**) NEL for TPLO. The radial osteotomy is centered on the midpoint of the tibial plateau line, and Ost is taken where the segment from IE meets that osteotomy. NEL = (B + C)/A. (**c**) Guénégo–Verwaerde index (GVI). MA (yellow), tibial mechanical axis; PJSL (red), patellar joint-surface length measured along the articular surface of the patella from the proximal to the distal pole; the green dashed line is the perpendicular dropped from the distal pole of the PJSL to the MA; D (blue) is the distance along the MA from its intersection with the tibial plateau to the foot of that perpendicular. GVI = D/PJSL. The red dashed outline and pale red shading in (a) indicate the resected cranially based wedge; the dashed extension of the MA in (c) is shown for reference only.

**Figure 3:**
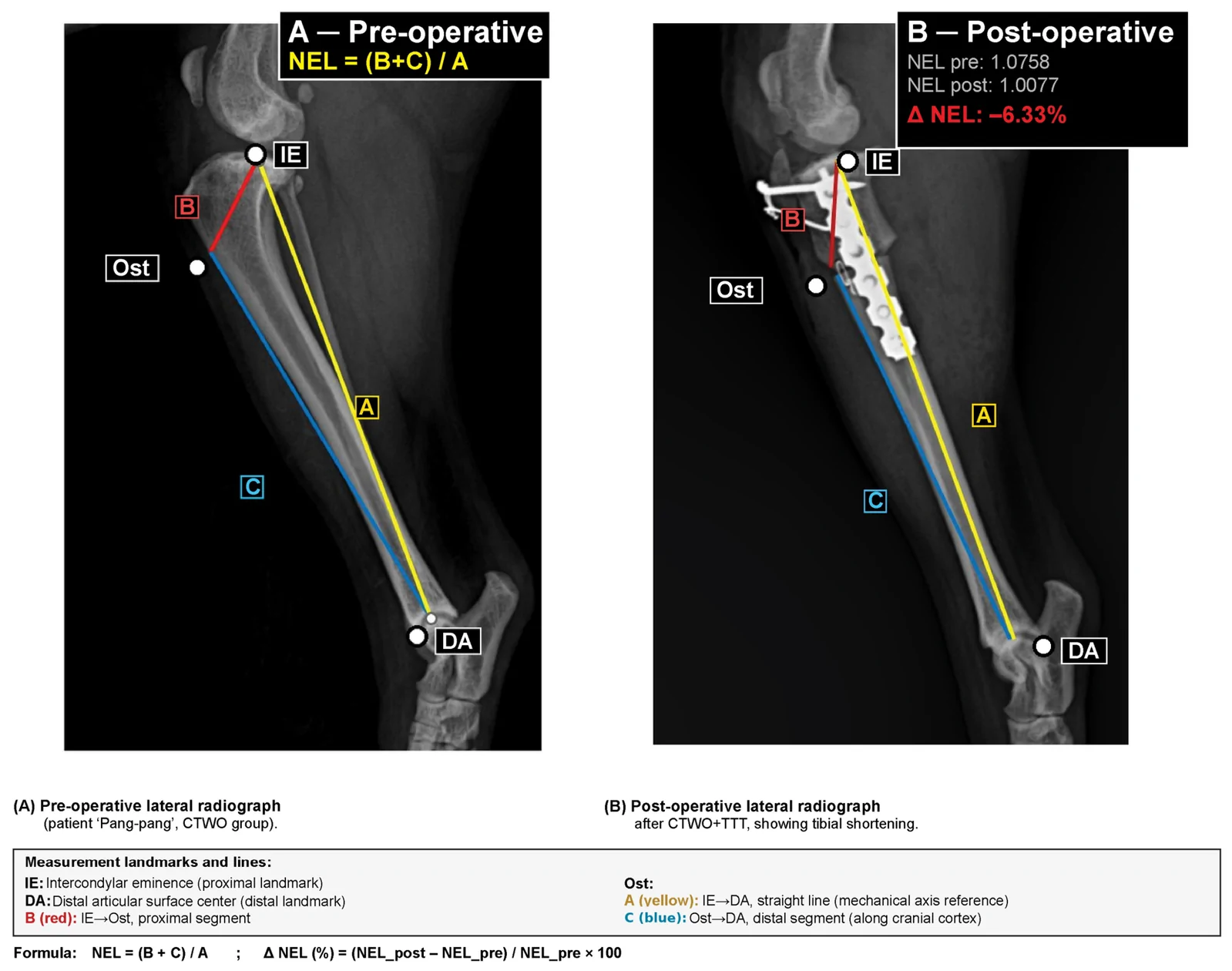
Measurement of the normalized effective tibial length (NEL) in a dog treated with CTWO + TTT (C08). (**A**) Preoperative mediolateral radiograph showing the three landmarks (IE, Ost and DA) and the three measurement lines A (yellow), B (red) and C (blue). (**B**) Immediate postoperative radiograph of the same stifle after CTWO + TTT, in which the osteotomy-induced tibial shortening is quantified (NEL 1.0758 → 1.0077, Δ −6.33%). IE, intercondylar eminence; Ost, intersection of the osteotomy line with the cranial cortex; DA, center of the distal articular surface; A, IE → DA (mechanical axis); B, IE → Ost (proximal segment); C, Ost → DA (distal segment, along the cranial cortex).

**Figure 4:**
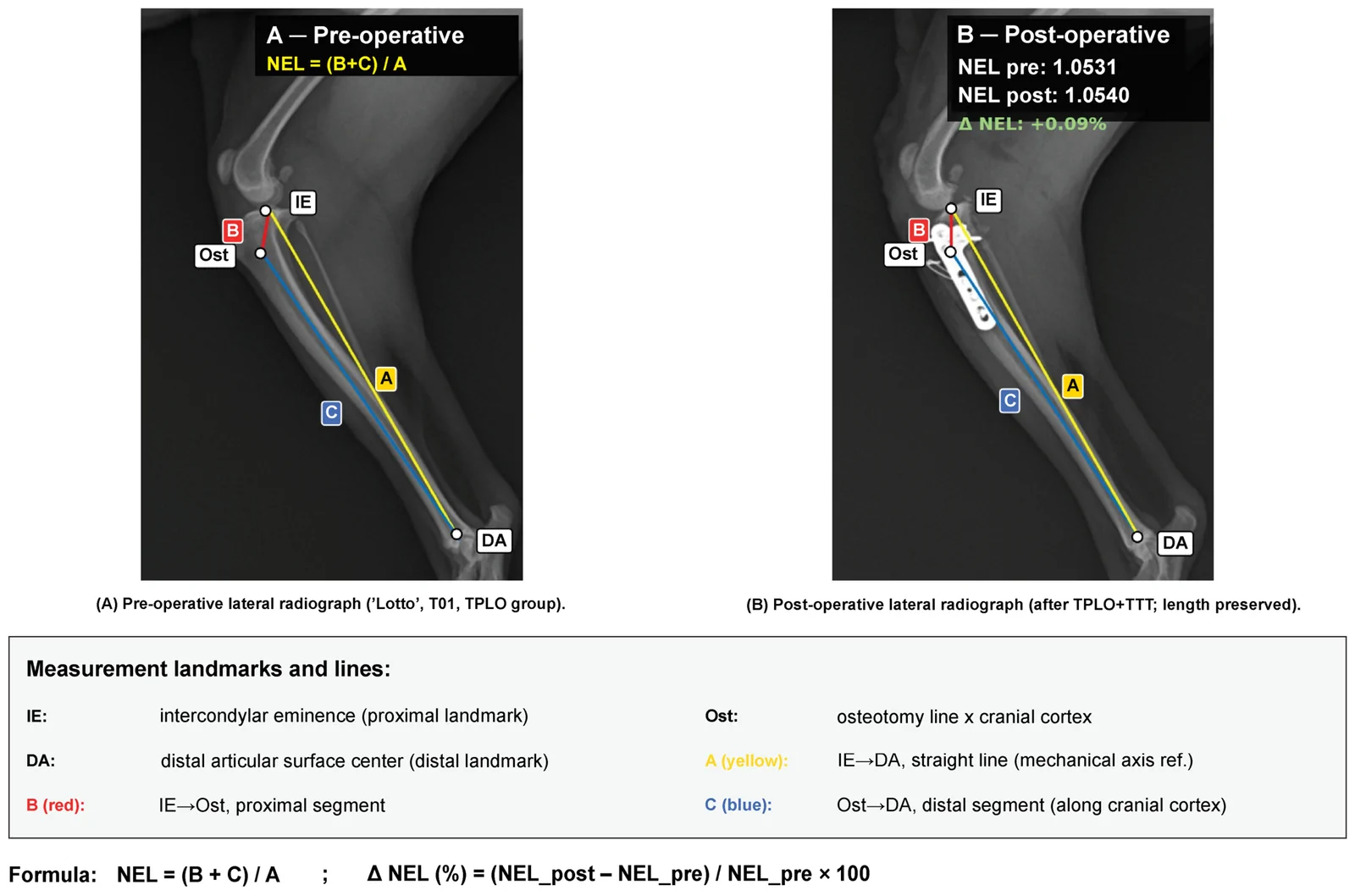
Measurement of the normalized effective tibial length (NEL) in a dog treated with TPLO + TTT (T01, right limb). (**A**) Preoperative and (**B**) immediate postoperative mediolateral radiographs of the same stifle showing the landmarks IE, Ost and DA and the lines A (yellow, IE → DA mechanical axis), B (red, IE → Ost proximal segment) and C (blue, Ost → DA distal segment). Because TPLO is a rotational osteotomy, the tibial length is preserved and the NEL changes negligibly (1.0531 → 1.0540, Δ +0.09%), in clear contrast to the tibial shortening seen after CTWO + TTT (Figure 3, Δ −6.33%).

To reduce measurement error caused by positional differences between radiographs, the NEL index was introduced and defined as NEL = (B + C)/A, where A is the straight IE → DA distance (the tibial mechanical-axis length, which is relatively stable to positioning), B is the straight IE → Ost distance (proximal segment), and C is the straight Ost → DA distance (distal segment). B + C is the segmental ETL, an effective length that also reflects postosteotomy tibial angulation. Normalization by A reduces the effects of radiographic magnification and positioning-dependent changes in straight-line distances. The change in NEL (NEL Δ%) was calculated as (NEL_post − NEL_pre)/NEL_pre × 100; negative and positive values indicate tibial shortening and tibial lengthening or reduced angulation, respectively. The landmarks and measurement lines are illustrated schematically in Figure 2a,b, and the method is shown on radiographs in Figure 3 (CTWO) and Figure 4 (TPLO). Because the osteotomy location differs between procedures, the absolute values of segments B and C, and thus NEL, are not directly comparable between groups. Therefore, the variable compared between groups was the within-limb relative change from the preoperative value (NEL Δ%), with each limb serving as its own reference. To our knowledge, this normalization has not previously been described.

#### 2.3.4. Guénégo–Verwaerde Index (GVI)

**Figure 5:**
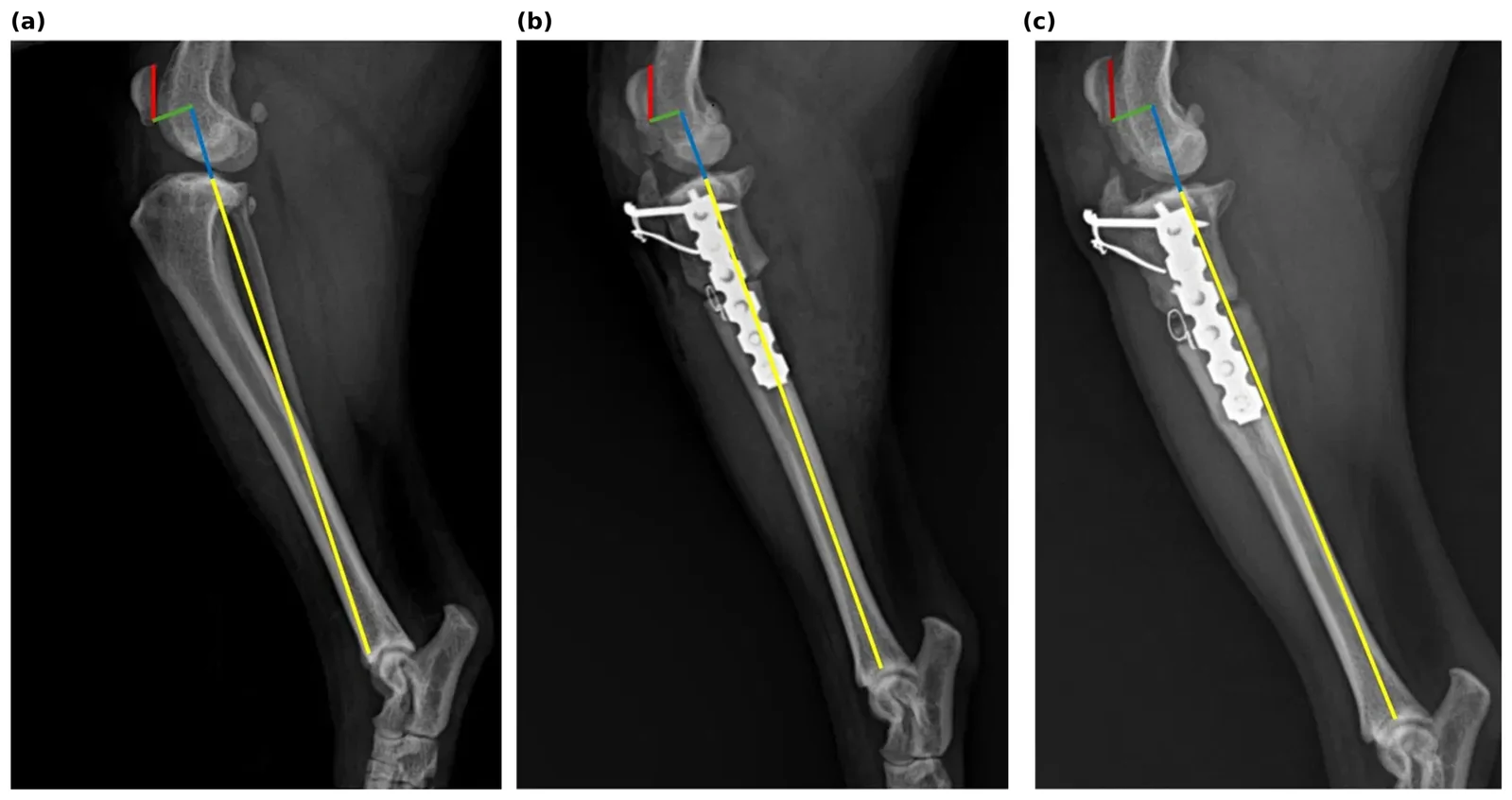
Measurement of the distance D for the Guénégo–Verwaerde index (GVI) in a dog treated with CTWO + TTT (C08). (**a**) Preoperative, (**b**) immediate postoperative, and (**c**) follow-up mediolateral radiographs of the same stifle. Yellow line, tibial mechanical axis (MA; intercondylar eminence → center of the distal articular surface); red line, patellar joint-surface length (PJSL), measured from the proximal to the distal pole of the patellar articular surface; green line, perpendicular dropped from the distal pole of the PJSL to the MA; blue line, distance D, measured along the MA from its intersection with the tibial plateau to the foot of the perpendicular. GVI = D/PJSL, and ΔGVI (%) = (GVI_post − GVI_pre)/GVI_pre × 100. In this stifle, the GVI decreased from 1.149 preoperatively to 1.079 immediately postoperatively (Δ −6.1%) and increased to 1.207 at follow-up (Δ +5.1%).

The patellar position was assessed using the GVI proposed by Guénégo and Verwaerde [16,17], defined as GVI = D/PJSL, where PJSL is the length of the caudal articular surface of the patella that contacts the trochlear groove (from the proximal to the distal pole) and D is the distance measured along the tibial mechanical axis (MA, identical to line A in this study) from the intersection of the MA and tibial plateau to the point at which a line through the distal patellar pole perpendicular to the MA meets the MA (Figure 2c and Figure 5). Guénégo and Verwaerde proposed the classification thresholds (GVI > 1.8, patella alta; 1.25–1.8, normal; 0.9–1.25, low; <0.9, patella baja); however, because no validated absolute threshold has been established for the canine stifle, absolute classification was not used as the primary measure. Instead, the patellar position was interpreted as the relative change from the preoperative value (GVI Δ%). One stifle (T02) was classified as grade IV MPL; in this dog, the luxation was not permanently fixed, and on all radiographs used for measurement, including the preoperative study, the patella lay within the trochlear groove, so that the PJSL and the distance D could be delineated in the same manner as in all other stifles. Measurements were made only on radiographs in which the patella was seated within the trochlear groove. Because the GVI is normalized to the PJSL, and the PJSL represents an anatomically fixed dimension that does not change with surgery, any large between-time-point discrepancy in the PJSL reflects differences in radiographic positioning or magnification rather than a true anatomical change. Stifles in which the PJSL differed by more than ±8% between time points were therefore excluded; this threshold was defined a priori by the authors as a conservative quality-control limit and was not derived from a previously published criterion. No stifle was excluded on this basis in the present series.

#### 2.3.5. Bone Healing Score (BHS)

Bone union at the osteotomy site was graded on follow-up radiographs using a five-point ordinal scale defined a priori for this study (bone healing score, BHS), based on the progressive disappearance of the osteotomy line and the extent of callus formation: 0, no sign of union (osteotomy line sharply defined, no callus); 1, early callus formation (osteotomy line clearly visible, minimal callus); 2, callus formation in progress (osteotomy line visible, callus bridging incomplete); 3, callus bridging with union in progress (osteotomy line becoming indistinct); and 4, complete union (osteotomy line obliterated, with continuity across the osteotomy). No standardized radiographic scoring system for osteotomy healing is currently established in veterinary orthopedics, and several different systems have been compared without a consensus emerging [18]; the present scale was therefore defined by the authors and has not been formally validated. The same predefined criteria were applied consistently to all stifles by a single, non-blinded observer; blinding was not feasible because the two osteotomy techniques are readily distinguishable on radiographs.

### 2.4. Statistical Analysis

The preoperative and postoperative GVI values within each group were compared using paired *t*-tests. Between-group comparisons of GVI Δ%, NEL Δ%, and bone healing score (BHS) were performed using Mann–Whitney U tests. The proportion of tibial shortening (Δ(B + C)) transferred to the patella was summarized descriptively as ΔD. Categorical variables, such as the complete union rate, were compared using Fisher’s exact test. The association between the magnitude of tibial plateau angle correction and the degree of tibial shortening within the CTWO + TTT group was assessed with the Spearman rank correlation coefficient. The distributions of continuous variables were assessed with the Shapiro–Wilk test, and non-parametric tests were used where normality could not be assumed. All tests were two-sided with α = 0.05. Analyses were performed using Python 3.11 (Python Software Foundation, Beaverton, OR, USA) with SciPy 1.11.

### 2.5. Use of AI-Assisted Technology

During the preparation of this manuscript, the authors used Claude (Anthropic; Opus and Sonnet models, 2026 releases) as an AI-assisted tool, accessed between May 2026 and August 2026. It was used to draft and refine the text of the manuscript, to assist with the retrieval and verification of published sources, to organize the authors’ dataset for analysis, and to render the schematic illustrations shown in Figure 1 and Figure 2 from designs specified by the authors. All clinical data, radiographic measurements and the underlying dataset were generated by the authors, and all statistical analyses were performed by the authors as described in Section 2.4. The authors checked each cited source against the original publication, and reviewed and edited all AI-assisted output. No generative AI tool was used to create, alter, or manipulate the original study data or clinical radiographs. The authors take full responsibility for the content of this publication.

## 3. Results

### 3.1. Animals

Twenty stifles from 18 dogs were included in this study (CTWO + TTT, 10 stifles; TPLO + TTT, 10 stifles). The mean body weights and ages in the CTWO and TPLO groups were 4.64 ± 1.45 kg and 8.1 ± 1.9 years, and 3.70 ± 1.32 kg and 6.7 ± 2.6 years, respectively; neither differed significantly between groups (*p* > 0.05). The MPL was predominantly grade III in both groups. Individual data are presented in Table 1. Preoperative TPA was 30.6 ± 3.0° in the CTWO + TTT group and 33.2 ± 2.3° in the TPLO + TTT group (Mann–Whitney U test, *p* = 0.048), and postoperative TPA was 6.4 ± 1.5° and 4.5 ± 1.3°, respectively (*p* = 0.009); the magnitude of correction was accordingly greater in the TPLO + TTT group (28.7 ± 2.8° versus 24.2 ± 2.8°, *p* = 0.006). Within the CTWO + TTT group, the magnitude of TPA correction, which corresponds to the angle of the resected wedge, was not correlated with the degree of tibial shortening (Spearman ρ = −0.24, *p* = 0.51). Two further stifles that met the diagnostic criteria were not eligible for analysis because implant failure required revision with double plating, precluding assessment of union at the planned time points. Both were treated using CTWO + TTT; no TPLO + TTT stifle required revision.

### 3.2. Tibial Length (NEL)

**Figure 6:**
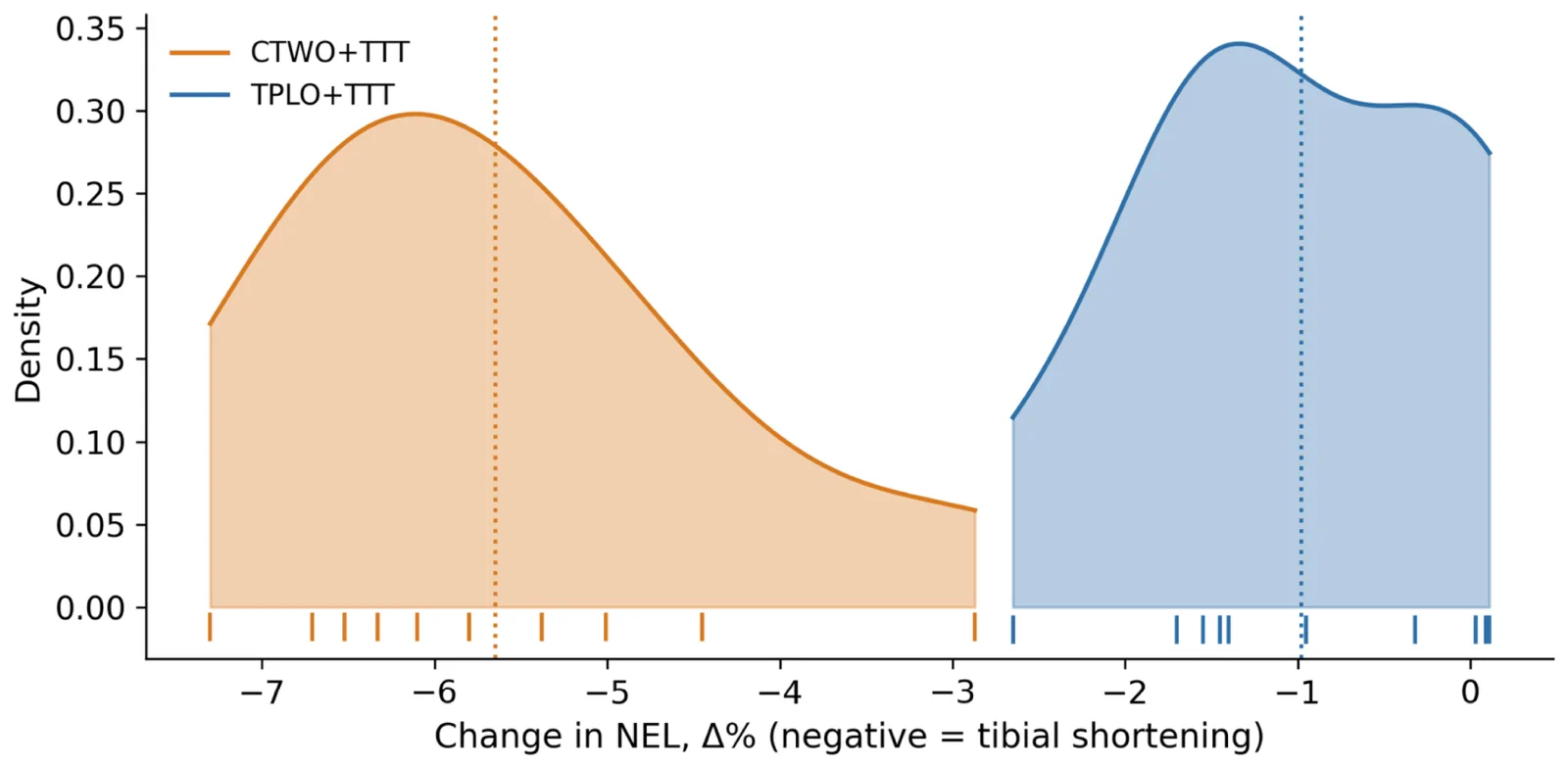
Distribution of the changes in NEL (Δ%) in the two groups. Kernel-density areas are filled only within each group’s observed range; rug ticks and dotted lines denote individual stifles and group means, respectively. The CTWO + TTT maximum (−2.87%) is smaller than the TPLO + TTT minimum (−2.65%); thus, the two distributions do not overlap in this sample (gap of 0.22 percentage points), separating tibial shortening (CTWO) from length preservation (TPLO) (Mann–Whitney U = 0, *p* < 0.001).

The mean NEL Δ% value was −5.65 ± 1.29% (ranging from −7.30 to −2.87%) in the CTWO + TTT group, with consistent shortening in every limb, and −0.98 ± 0.93% (ranging from −2.65 to +0.11%) in the TPLO + TTT group, in which tibial length was essentially preserved. The between-group difference was highly significant (Mann–Whitney U = 0, *p* < 0.001). The two NEL Δ% distributions did not overlap in this sample (Figure 6; the CTWO maximum of −2.87% was smaller than the TPLO minimum of −2.65%), and the variance in the TPLO group was 1.9-fold smaller than that in the CTWO group. Individual measurements are presented in Table 2. Segmental analysis showed that the shortening after CTWO + TTT occurred almost entirely in the distal segment: ΔC was −5.82 ± 2.61 mm whereas ΔB was −0.03 ± 0.25 mm; therefore, the distal segment accounted for a mean of 99.8% of the total change in B + C (range 93.9–107.2%). In the TPLO + TTT group both segments were largely unchanged (ΔB +0.01 ± 0.15 mm; ΔC −0.42 ± 0.80 mm).

### 3.3. Patellar Position (GVI)

**Figure 7:**
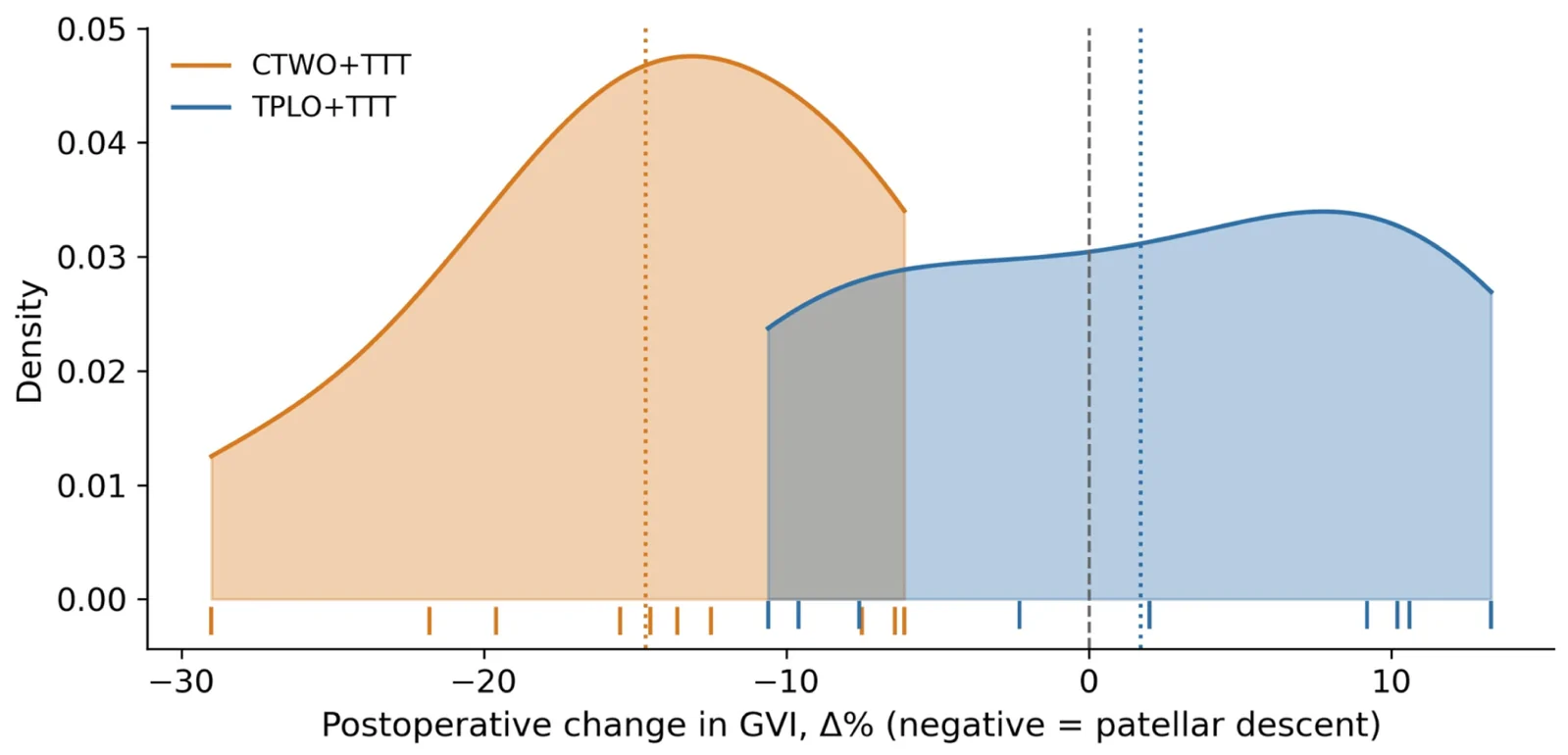
Distribution of the changes in the postoperative patellar position (GVI Δ%). Kernel-density areas are filled only within each group’s observed range; rug ticks denote individual stifles, dotted lines indicate the group means, and the vertical dashed line (0%) indicates no change (negative, descent; positive, elevation). CTWO + TTT stifles lie entirely in the negative range (mean: −14.6%), whereas TPLO + TTT stifles cluster around 0% (mean: +1.7%). In contrast to NEL, the two distributions partially overlapped (−10.6 to −6.1%), but their centers were clearly separated (Mann–Whitney U test, *p* = 0.002).

Patellar position (GVI) was analyzed in 20 stifles. In the CTWO + TTT group, postoperative GVI decreased in all 10 stifles; from a preoperative mean of 1.141 to a postoperative mean of 0.973 (mean change −14.6 ± 7.3%, paired *t*-test *p* < 0.001), indicating that the patella descended relative to the tibial plateau immediately after osteotomy and reduction. In the TPLO + TTT group, the mean GVI was unchanged (1.048 → 1.058; mean change, +1.7 ± 9.0%, paired *t*-test *p* = 0.73; only 4 of 10 stifles showed mild descent). The postoperative patellar descent was significantly smaller after the TPLO + TTT procedure (Mann–Whitney U test, *p* = 0.002; Figure 7). Moreover, the patellar descent in the CTWO + TTT group (ΔD, 1.52 mm) was only approximately 26% of the tibial shortening (Δ(B + C), 5.85 mm); thus, a large part of the tibial shortening was not transferred to the patellar position. At follow-up, the CTWO + TTT group showed partial recovery (−9.5%) whereas the TPLO + TTT group maintained its position (+3.5%). Individual GVI values and patterns are listed in Table 3.

### 3.4. Patterns of Patellar-Position Change

Postoperative and follow-up patterns of patellar position change were analyzed (Table 4). All 10 CTWO + TTT stifles showed postoperative descent. At follow-up, 6 of 10 stifles showed a tendency to recover (follow-up GVI > postoperative GVI), and stifles in 4 of 10 patients (C01, C02, C04, and C09) continued to descend throughout follow-up. In contrast, the TPLO + TTT group showed a mixture of postoperative descent and elevation (descent in 4/10, elevation or maintenance in 6/10) with no consistent direction, and on average, the patellar position was slightly elevated at follow-up (+3.5%). Thus, CTWO + TTT consistently lowered the patella in proportion to tibial shortening, whereas TPLO + TTT preserved the tibial length and produced small, stable postoperative changes in the patellar position, indicating a relative advantage of TPLO + TTT for the preservation of the patellar position. For clarity, “recovery” denotes an increase in GVI between the immediate postoperative and follow-up examinations, indicating a more proximal patellar position relative to the tibial plateau at follow-up than immediately after surgery. This likely reflects re-adaptation of the extensor mechanism and surrounding soft tissues rather than a change in tibial length, which remains fixed after stabilization. “Sustained descent” denotes a GVI that remained below the preoperative value and did not increase at follow-up.

### 3.5. Overall Comparison Between Groups

**Figure 8:**
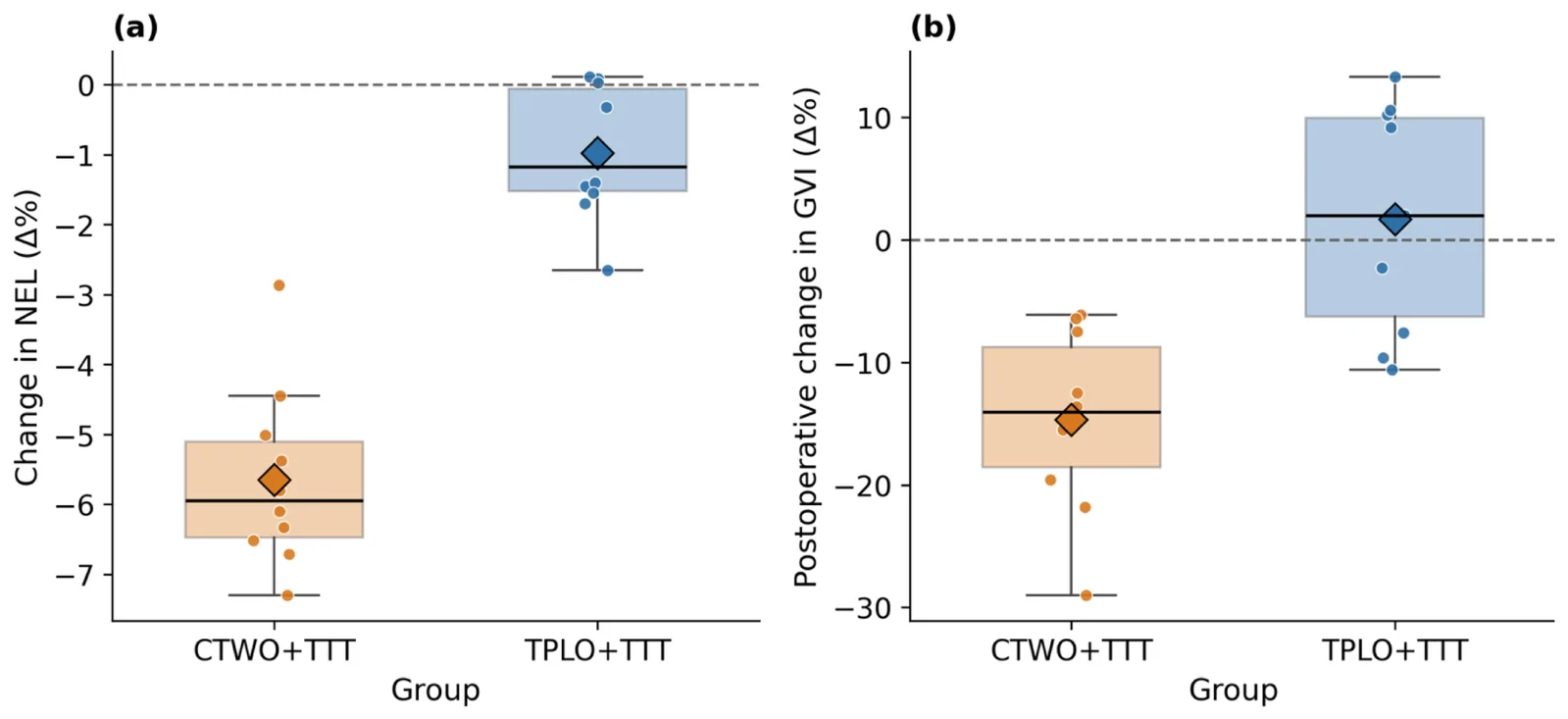
Box plots comparing the radiographic outcomes of the two procedures: Changes in (**a**) NEL (Δ%) and (**b**) GVI (Δ%) in the CTWO + TTT (*n* = 10) and TPLO + TTT (*n* = 10) groups. Points represent individual stifles, diamonds indicate group means, and the dashed line indicates no change (0%). CTWO + TTT consistently shortened the tibia (mean −5.65%) and lowered the patella (mean −14.6%), whereas TPLO + TTT preserved both at approximately 0% (NEL −0.98%, GVI + 1.7%). Between groups: NEL Mann–Whitney U test *p* < 0.001; GVI *p* = 0.002.

Radiographic union was assessed at 8–10 weeks. In the TPLO + TTT group, the mean BHS was 3.90 ± 0.32 (BHS 4 in 9 of 10 stifles; BHS 3 in one). In the CTWO + TTT group, the mean BHS was 2.70 ± 0.48 (BHS 3 in 7 of 10 stifles; BHS 2 in three). No stifle in either group was graded BHS 0 or 1. Callus formation with union in progress was present in every stifle of both groups, and no non-union was observed. The principal radiographic outcomes are summarized in Table 5. CTWO + TTT and TPLO + TTT differed significantly in terms of the NEL Δ%, BHS, complete union rate (all *p*-values < 0.001), and postoperative GVI Δ% (*p* = 0.002). The changes in NEL and GVI are shown as box plots in Figure 8.

## 4. Discussion

### 4.1. Principal Findings

In small-breed dogs undergoing single-session correction of concurrent CrCL rupture and MPL, the choice of proximal tibial osteotomy determined the geometric starting point for the concurrent TTT. CTWO shortened the tibia in every limb and lowered the patella in every limb, whereas TPLO preserved both; the two distributions of tibial-length change did not overlap in this sample. The patellar descent after CTWO + TTT amounted to only about a quarter of the tibial shortening, indicating that tuberosity proximalization compensated substantially but not completely. Radiographic union was further advanced after TPLO + TTT at the 8–10-week assessment, although union was progressing in every stifle of both groups. The findings that follow directly from osteotomy geometry—tibial-length preservation and maintenance of the patella–tibial plateau relationship—were the most consistent; the healing difference is more difficult to attribute to technique alone, for the reasons discussed below.

### 4.2. Tibial Length: Comparison with Reported Geometric Consequences of Wedge Osteotomy

The tibial shortening observed after CTWO + TTT (NEL Δ% −5.65 ± 1.29%; mean Δ(B + C) 5.85 mm) is a direct consequence of removing a cranially based wedge and apposing the cut surfaces, and its magnitude can be compared with previously reported values. Bailey et al. analyzed the geometry of tibial wedge osteotomy and showed that tibial shortening is an unavoidable consequence of the procedure, increasing in proportion to the wedge angle [12]. Guénégo et al., using a modified cranial closing wedge osteotomy planned on the tibial anatomical–mechanical axis angle in large dogs with TPA > 30°, reported a median tibial shortening of 3.5 mm (range 1–7.6 mm), comparable with the mean difference of 2.5–3 mm documented for closing wedge osteotomy in earlier publications [16].

Our mean shortening of 5.85 mm is therefore larger in absolute terms than these reported values, despite our patients being small-breed dogs (mean body weight 4.64 kg in the CTWO + TTT group) rather than large dogs. Two factors plausibly account for this. First, the technique differs: Guénégo et al. deliberately modified the standard procedure to minimize the wedge, positioning its apex at the caudodistal insertion of the medial collateral ligament rather than at the caudal tibial cortex and limiting the correction to 65% of the preoperative TPA, whereas the present series used a standard cranial tibial wedge osteotomy. Second, and more importantly for clinical interpretation, the same absolute shortening represents a far greater proportional loss in a small-breed tibia: a 5.85 mm shortening in a 4 kg Maltese is not equivalent to the same value in a 35 kg Labrador. This is precisely why we expressed shortening as a normalized, dimensionless change rather than in millimeters. Notably, within the CTWO + TTT group, the angle of correction was not correlated with the degree of shortening, which is consistent with this geometric reasoning: for a given wedge angle, the length of bone removed at the cranial cortex depends on the width of the tibia, so that angle alone does not predict the resulting shortening.

The near-zero change in the TPLO + TTT group agrees with the theoretical prediction that a radial osteotomy with rotation of the proximal fragment preserves tibial length, and provides internal validation that the NEL index behaves as intended: it detects the shortening that geometry predicts must occur, and does not detect shortening where geometry predicts none. Segmental analysis further showed that the shortening was confined almost entirely to the distal segment (ΔC accounting for 99.8% of the total change), as the geometry of a cranially based wedge predicts, so that the proximal geometry linking the tibial plateau, the osteotomy and the tuberosity was not substantially disturbed by the osteotomy itself.

### 4.3. Patellar Position: Agreement with Previously Reported GVI Changes

The magnitude of patellar descent we observed after CTWO + TTT corresponds closely to that reported in the only comparable study using the same index. Guénégo et al. reported a median GVI decrease from 1.59 on the unaffected contralateral side to 1.35 postoperatively after modified closing wedge osteotomy—a relative reduction of approximately 15% [16]. In the present series, the mean GVI in the CTWO + TTT group fell from 1.141 preoperatively to 0.973 immediately postoperatively, a relative change of −14.6%. The close agreement between these two independently derived values, in different breeds, body sizes and surgical variants, supports the validity of our measurements and indicates that the proportional patellar descent after wedge osteotomy is a reproducible finding. The mechanism is clarified by the ex vivo work of Shimada et al., who compared TPLO and closing wedge ostectomy in the same dogs and showed that the wedge procedure displaces the origin of the patellar ligament distally and exerts significantly greater downward traction on the patella, whereas TPLO does not displace that origin [15].

The absolute values, however, warrant caution. The published classification of GVI (>1.8 alta; 1.25–1.8 normal; 0.9–1.25 low; <0.9 patella baja) has not been validated for small-breed dogs, and in the present series the absolute GVI is additionally influenced by the wide range of stifle flexion angles used, as described in the Methods. We therefore do not classify our stifles by these thresholds and do not claim that any stifle developed patella baja. The proportional descent after wedge osteotomy was comparable to previous reports. However, small-breed dogs with concurrent MPL had a lower baseline patellar height, so the same proportional descent consumed a larger fraction of the available margin. Whether this translates into clinically relevant patella baja in this population cannot be determined from the present data and requires prospective study with functional outcome measures.

### 4.4. Patellar Position After TPLO + TTT and the Limits of the GVI

For the TPLO + TTT group, our finding of an essentially unchanged GVI (+1.7%, *p* = 0.73) is consistent with the mechanism described above: because TPLO rotates the proximal fragment without altering the relationship between the patella and the tibial plateau, the GVI would be expected to remain within its preoperative range after this procedure. Only 4 of 10 stifles descended postoperatively, the remainder being preserved or slightly elevated, with no consistent directional tendency, and at follow-up the group maintained its position (+3.5%) whereas the CTWO + TTT group only partially recovered (−9.5%) and remained below its preoperative value. Our findings therefore confirm the expected behavior of the index under TPLO rather than indicating that patellar position is entirely unaffected. The same authors who described the GVI reported a decrease in the Insall–Salvati-type ratio after TPLO, reflecting a reduction in the distance between the distal patellar pole and the tibial crest, which the GVI is not designed to capture [15,16]. As noted in the Limitations, we did not measure that index, and our conclusions regarding patellar position are confined to the patella–tibial plateau relationship.

Our choice of the relative change in GVI, rather than its absolute value, as the primary measure of patellar position also warrants explanation here. Three considerations informed it. First, no validated absolute threshold exists for classifying the canine GVI as baja, normal, or alta, so an absolute classification cannot serve as a reliable primary measure. Second, a relative change compares the preoperative and postoperative states of the same limb and is therefore unaffected by between-individual variation in patellar size and anatomy, reflecting more directly the effect of the procedure itself. Third, because the GVI is the ratio of D to PJSL, the magnification effect within a single radiograph is inherently removed, which makes the index suitable for comparisons between time points. Accordingly, the immediate postoperative and follow-up GVI Δ% values were used as measures of the immediate surgical effect and of soft-tissue re-adaptation over time, respectively.

### 4.5. Bone Healing in the Context of Reported Healing Times

Complete radiographic union at the 8–10-week assessment was observed in 9 of 10 TPLO + TTT stifles but in none of the CTWO + TTT stifles. This difference requires careful interpretation, because the assessment window is early relative to the healing times reported for wedge osteotomies, and because it does not represent a failure of union: every CTWO + TTT stifle showed callus formation with union in progress (mean BHS 2.70 ± 0.48; range 2–3), and none was graded as showing no sign of union or early callus only. In the most directly comparable study, Carrera et al. reported a mean time to bone healing of 70.2 ± 21.2 days after combined total proximal tibial osteotomy and closing wedge ostectomy in dogs with concomitant MPL and cranial cruciate ligament disease [19]. Our assessment at 8–10 weeks (56–70 days) therefore coincides with, or slightly precedes, the mean healing time reported for this procedure in an equivalent population, so that incomplete union in the CTWO + TTT group at this time point is the expected finding rather than an adverse one. Leonard et al., who first described the combination of TPLO with TTT, documented union of both osteotomies at nine weeks postoperatively [20], a timeframe consistent with the union observed in our TPLO + TTT group.

Two further considerations apply. First, a technique-intrinsic factor plausibly contributes: the TPLO radial osteotomy is performed in the proximal tibial metaphysis, a region rich in cancellous bone with abundant vascularity, whereas the cranial tibial wedge osteotomy is performed more distally, where the cortical component is proportionally greater; cancellous bone unites more rapidly than cortical bone, and this difference in osteotomy location, independent of operator experience, provides a biological basis for faster radiographic union after TPLO + TTT. This consideration was in fact one reason for the surgeon’s transition from CTWO to TPLO during the study period. Second, however, Oxley et al., comparing 97 TPLO with 74 modified closing wedge osteotomy procedures assessed at 8 weeks, found comparable complication rates and clinical outcomes between the techniques when performed by surgeons experienced with them [21]—a qualification directly relevant to the present series, in which the CTWO + TTT procedures were performed earlier in the study period. As noted in the Results, two further CTWO + TTT stifles were not eligible for analysis because implant failure required revision, whereas no TPLO + TTT stifle required revision; the healing data reported here therefore describe stifles in which fixation remained intact and do not represent the complication rate of either procedure, which has been documented for both techniques in several larger series [22,23,24,25,26,27]. The difference observed here is best characterized as a difference in the rate of radiographic union at a fixed early time point, not as a difference in the ultimate capacity for union.

### 4.6. Methodological Contribution of the NEL Index

Quantifying the length consequences of a proximal tibial osteotomy is complicated by the fact that the measurement itself is sensitive to limb positioning. Previous studies have reported tibial shortening after wedge osteotomy in absolute terms, which is informative but carries two limitations. First, absolute values cannot be compared across body sizes, since the same millimeter value represents different proportions of the tibia in a small- and large-breed dog. Second, a straight-line effective tibial length measured segmentally across the osteotomy varies with radiographic magnification and with the degree of stifle flexion, both of which are difficult to standardize in a retrospective clinical series. The NEL index addresses both problems by normalizing the segmental length to the straight intercondylar-eminence-to-distal-articular-center distance, which represents the tibial mechanical axis and is comparatively stable across projections of the same limb. In the present data, the reference length A changed by a mean of 1.26% between time points (range 0.03–4.76%), and remained within ±1.5% in 14 of the 20 stifles. It should be acknowledged, however, that in a minority of limbs A varied by up to approximately 4.8%, which is of the same order as the differences being measured; in two TPLO + TTT stifles (T04 and T06) the segmental length B + C in fact increased slightly while A increased more, so that the calculated NEL Δ% was negative. Normalization therefore reduces, but does not eliminate, the influence of positioning, and this residual variability is acknowledged in the Limitations. Because NEL is dimensionless, it is directly comparable across body sizes, and because the primary measure is the within-limb relative change, magnification and positioning effects common to the numerator and denominator are largely offset. The index is applicable beyond the specific comparison reported here: any procedure that alters tibial length, including modified wedge techniques designed to minimize shortening, could be characterized on the same dimensionless scale, allowing results from different breeds and body sizes to be compared directly. It is not, however, insensitive to all sources of error: it corrects for effects that scale the whole image or the whole limb, but not for measurement error at individual landmarks, and it was applied here by a single observer without a reproducibility analysis.

### 4.7. The Present Findings in the Context of Combined-Lesion Surgery

Studies of concurrent CrCL rupture and MPL have focused predominantly on complication rates and clinical function rather than on the geometric consequences of the chosen osteotomy, and the reported rates of recurrent patellar luxation differ appreciably between approaches. Fauron et al. reported complication rates of up to 46.9% after extracapsular stabilization combined with TTT, of which 15.6% were major complications requiring revision, most commonly recurrent patellar luxation, infection, and implant failure [7]. Carrera et al. achieved initial patellar and stifle stability in 87.1% of cases but recorded patellar reluxation requiring surgical revision in 12.9% [19]. By contrast, Redolfi and Grand, reporting 24 stifles treated by TPLO combined with TTT for grade III or IV MPL, recorded recurrent luxation in a single stifle (4.2%), the remaining major complications being surgical site infection [28]; and Jung and Kang, in nine stifles of small-breed dogs with grade IV MPL treated by a modified TPLO with TTT, observed no major complications and no reluxation [29]. Larger series using modified TPLO techniques for the same combined lesion have also been reported [30,31]. These clinical series are heterogeneous in case selection, fixation and follow-up, and no direct statistical comparison between them is possible; nevertheless, recurrent patellar luxation emerges as a recognized failure mode that appears less frequent after rotational than after wedge-based procedures.

What has not previously been quantified is the mechanism by which the underlying osteotomy might predispose to reluxation. The present study addresses that gap: by measuring tibial shortening and the resulting change in patellar position on a dimensionless scale, it provides a geometric account of why maintaining patellar tracking is mechanically more demanding after a wedge osteotomy than after a rotational one. Our observation that only about a quarter of the tibial shortening was translated into patellar descent, and that four of ten CTWO + TTT stifles nevertheless showed sustained descent through follow-up, indicates that tuberosity proximalization compensates substantially but not reliably. These findings are therefore complementary to, rather than in conflict with, the existing outcome literature: they describe the anatomical starting point on which those clinical outcomes are built, and they suggest that comparisons of complication rates between techniques should consider the differing geometric burden placed on the tuberosity correction. It should be noted that a trochlear block recession was performed in every stifle in the present series, whereas trochleoplasty was applied selectively in some previous reports [20]; the patellar-position differences we observed therefore arose despite uniform trochlear deepening in both groups.

### 4.8. Overall Synthesis

The differences observed between the two procedures fall into two categories, which warrant different levels of interpretive confidence. Differences attributable to osteotomy geometry come first. Tibial-length preservation is a direct and unavoidable consequence of the geometry of each osteotomy: in TPLO the proximal fragment is rotated about a radial cut, so that the cut surfaces travel along an arc and the absolute tibial length is theoretically unchanged, whereas in CTWO a cranially based wedge is removed and the surfaces are apposed, so that the tibia is necessarily shortened. The observed NEL Δ% values (−0.98% versus −5.65%, *p* < 0.001), with non-overlapping ranges in this sample, correspond closely to this geometric prediction. This difference is intrinsic to the techniques and is not expected to be altered by operator experience, and the consistent patellar descent observed in every CTWO + TTT stifle follows from the same mechanism, since tibial shortening reduces the working length of the extensor mechanism.

In contrast, the magnitude of the between-limb variability, the degree of residual patellar descent after tuberosity proximalization, and the difference in the rate of radiographic union depend on osteotomy geometry and surgical execution—wedge sizing, apposition and compression of the cut surfaces, and the extent to which the tuberosity could be proximalized. Because the CTWO + TTT procedures in this series were performed earlier in the surgeon’s experience than the TPLO + TTT procedures, these differences should be interpreted as the combined effect of technique and operator experience rather than as an isolated property of the osteotomy method. The present data do not indicate that CTWO inherently precludes bone union; rather, union at 8–10 weeks was slower and less complete after CTWO + TTT under the conditions of this study.

For the specific goals of preserving tibial length and maintaining the patella–tibial plateau relationship in small-breed dogs with concurrent CrCL rupture and MPL, TPLO + TTT therefore offers a structural advantage that does not depend on operator experience, and it places a smaller corrective burden on the surgeon, because lateral transposition alone is generally sufficient whereas CTWO additionally requires proximalization of the tuberosity to compensate for shortening—a correction that is anatomically constrained and cannot always be achieved in full.

### 4.9. Limitations

This study had several limitations. First, the retrospective study included a small sample of twenty stifles (10 per group). Two dogs underwent bilateral surgery with their limbs treated as independent units of analysis; limbs from the same dog are not strictly independent, which may have underestimated variance. In addition, cases were selected from a larger surgical population based on diagnostic criteria and radiographic completeness rather than consecutively, so selection bias cannot be excluded. All procedures were performed by a single surgeon at a single institution, so multicenter, multi-surgeon studies are required to establish generalizability.

Second, all measurements were made by a single, non-blinded observer, and no reproducibility analysis (e.g., an intraclass correlation coefficient) was performed. Blinding was not feasible because the two osteotomy techniques are readily distinguishable on radiographs and grading was performed by the operating surgeon. To limit subjectivity, a predefined criterion (progressive disappearance of the osteotomy line) was applied consistently to all stifles. Observer bias nevertheless cannot be excluded.

Third, radiographic positioning was not uniform. The stifle flexion angle ranged from approximately 110° to 140°, reflecting the imaging priority of this cohort, in which the mediolateral projection served primarily as a tibial compression stress view for cruciate assessment; the distribution of angles was comparable in the two groups, so that positioning did not differ systematically between them, but the range exceeds that over which patellar height indices have been validated, and condylar superimposition was imperfect in some stifles. Absolute GVI and NEL values were therefore not compared between groups. Instead, between-group comparisons used within-limb relative changes, with each limb radiographed identically across time points, thereby largely, though not entirely, minimizing the effect of flexion angle. Residual within-limb variation nevertheless remains a source of measurement noise: the normalizing reference length A itself varied by a mean of 1.26% between time points (range 0.03–4.76%), so that normalization reduces but does not eliminate the influence of positioning.

Fourth, the bone healing comparison requires particular caution. The BHS scale was defined by the authors and has not been validated, as no standardized radiographic scoring system for osteotomy healing is established in veterinary orthopedics; furthermore, limb positioning is known to substantially influence osteotomy healing scores [18], and although the distribution of flexion angles was comparable in the two groups, positioning was not standardized. Importantly, no non-union or failure of union occurred in either group. All CTWO + TTT stifles showed callus formation with union in progress, indicating that the between-group difference represents a difference in the rate of radiographic union at an early point rather than in the capacity for union. Follow-up was also short: radiographs were obtained at 3–4 and 8–10 weeks, and all outcome measures were assessed at the 8–10-week examination, so that ultimate union and long-term patellar stability were not evaluated, and no dynamic or functional outcomes (gait, pain, and owner assessment) were obtained. Patellar position was assessed using the GVI alone, which references the patella to the tibial plateau and does not detect changes in the patella-to-tibial-crest relationship; the GVI also requires the patella to be visualized within the trochlear groove and cannot be applied reliably to a permanently fixed luxation.

Fifth, the two groups were not balanced with respect to baseline tibial geometry: the preoperative TPA was significantly higher in the TPLO + TTT group. Because a steeper tibial plateau generally represents a more demanding correction, this difference is unlikely to have favored the TPLO + TTT group, but the two groups were not directly equivalent at baseline. In addition, the CTWO + TTT procedures were performed earlier in the study period than the TPLO + TTT procedures, and differences in surgical execution over time cannot be formally separated from differences attributable to the technique itself.

## 5. Conclusions

This retrospective study quantitatively compared the radiographic outcomes of TPLO + TTT and CTWO + TTT, using the NEL and GVI, in small-breed dogs with concurrent CrCL rupture and MPL. CTWO + TTT consistently shortened the tibia (NEL Δ% −5.65% versus −0.98%, *p* < 0.001; non-overlapping ranges in this sample) and lowered the patella in every stifle (10/10; mean GVI Δ% −14.6%). By contrast, TPLO + TTT preserved tibial length and the patella–tibial plateau relationship (GVI Δ% +1.7%, *p* = 0.73; between-group *p* = 0.002). These findings correspond closely with the geometric prediction that a rotational osteotomy preserves length whereas a wedge osteotomy does not, reflecting an inherent difference between the two techniques. The proportional patellar descent observed after CTWO + TTT is closely comparable to that previously reported after modified closing wedge osteotomy in large dogs; however, because small-breed dogs with concurrent MPL have a lower baseline patellar height, the same proportional descent consumes a larger fraction of the available margin. Patellar descent amounted to only approximately 26% of the tibial shortening, indicating substantial but incomplete compensation by tuberosity proximalization, which is anatomically limited in extent.

Radiographic union at 8–10 weeks appeared more advanced after TPLO + TTT and between-limb variability was smaller. This observation should, however, be regarded as preliminary: the assessment window coincides with the mean healing time reported for wedge osteotomies in comparable populations, the CTWO + TTT cases were performed earlier in this single surgeon’s experience, radiographic positioning was not standardized, which is known to influence healing scores, and the grading scale was author-defined and non-blinded. Importantly, no non-union occurred in either group; union was in progress in every stifle. The finding is consistent with the greater cancellous bone content at the proximal metaphyseal TPLO site, but the present data do not establish a difference in the ultimate capacity for union between the two techniques.

Taken together, TPLO + TTT provided a more predictable outcome of tibial length and the patella–tibial plateau relationship—features directly governed by osteotomy geometry—and required less demanding tuberosity correction. The methodological contribution of this study is the NEL index, which normalizes the segmental effective tibial length to the tibial mechanical axis and is therefore comparatively robust to differences in radiographic positioning and directly comparable across body sizes. Multicenter, multi-surgeon studies with blinded assessment, reproducibility analysis, and long-term clinical follow-up are warranted to determine which of the differences observed here are attributable to the techniques themselves.

## Figures and Tables

**Table 1:** Signalment and procedure for the 20 stifles.

ID	Procedure	Limb	Breed	Weight (kg)	Age	Sex	MPL	BHS	Pre-op TPA (°)	Post-op TPA (°)
C01	CTWO	R	Yorkshire Terrier	4.00	9 y	F	III	3	27	5
C02	CTWO	L	Maltese	5.00	8 y	F	III	3	32	6
C03	CTWO	L	Maltese	4.00	10 y	M	III	3	30	8
C04	CTWO	R	Poodle	4.40	6 y	M	II	2	26	5
C05	CTWO	L	Spitz	7.30	6 y	F	II	3	36	7
C06	CTWO	R	Maltese	4.04	5 y	M	III	2	34	8
C07	CTWO	L	Maltese	2.40	11 y	F	II	3	31	6
C08	CTWO	R	Maltibichon	6.90	8 y	M	II	2	30	5
C09	CTWO	R	Maltese	4.20	9 y	CM	III	3	29	9
C10	CTWO	L	Maltese	4.20	9 y	CM	III	3	31	5
T01	TPLO	R	Poodle	5.30	11 y	M	III	4	35	6
T02	TPLO	R	Maltese	3.40	9 y	F	IV	4	33	5
T03	TPLO	L	Maltese	4.90	5 y	M	III	4	31	7
T04	TPLO	R	Pomeranian	3.85	5 y 5 m	SF	III	3	37	4
T05	TPLO	R	Bichon Frise	3.48	4 y 7 m	SF	III	4	33	4
T06	TPLO	L	Maltese	3.10	4 y 2 m	SF	II	4	35	3
T07	TPLO	R	Maltese	5.70	6 y 7 m	SF	III	4	32	3
T08	TPLO	R	Bichon Frise	3.60	3 y 7 m	SF	III	4	35	4
T09	TPLO	R	Maltese	1.82	9 y	SF	III	4	31	5
T10	TPLO	L	Maltese	1.82	9 y	SF	III	4	30	4

**Table 2:** NEL measurements (mm).

ID	Limb	A_pre	A_post	B_pre	B_post	C_pre	C_post	NEL_pre	NEL_post	Δ%	ΔB (mm)	ΔC (mm)	ΔA (%)
C01	R	84.19	84.37	18.14	18.11	72.15	66.85	1.0725	1.0070	−6.10	−0.03	−5.30	+0.21
C02	L	68.94	69.88	16.17	16.04	58.72	54.33	1.0863	1.0070	−7.30	−0.13	−4.39	+1.36
C03	L	71.03	69.40	18.00	17.93	58.40	52.39	1.0756	1.0133	−5.80	−0.07	−6.01	−2.29
C04	R	102.42	97.54	18.30	18.05	91.76	79.93	1.0746	1.0045	−6.52	−0.25	−11.83	−4.76
C05	L	87.10	85.99	23.10	23.63	70.16	62.26	1.0707	0.9988	−6.71	+0.53	−7.90	−1.27
C06	R	76.84	74.70	15.75	15.35	66.70	60.49	1.0730	1.0153	−5.38	−0.40	−6.21	−2.79
C07	L	62.53	62.86	16.71	16.79	48.97	45.93	1.0504	0.9978	−5.01	+0.08	−3.04	+0.53
C08	R	95.63	96.41	21.71	21.60	81.17	75.55	1.0758	1.0077	−6.33	−0.11	−5.62	+0.82
C09	R	90.86	89.90	17.73	17.84	78.25	72.90	1.0564	1.0093	−4.45	+0.11	−5.35	−1.06
C10	L	89.00	89.11	17.27	17.28	76.24	73.66	1.0507	1.0205	−2.87	+0.01	−2.58	+0.12
T01	R	100.25	100.31	11.04	10.89	94.53	94.84	1.0531	1.0540	+0.09	−0.15	+0.31	+0.06
T02	R	78.22	78.05	9.52	9.64	72.72	72.16	1.0514	1.0480	−0.32	+0.12	−0.56	−0.22
T03	L	77.54	77.56	10.32	10.50	71.60	70.25	1.0565	1.0411	−1.45	+0.18	−1.35	+0.03
T04	R	61.73	63.66	9.38	9.38	55.81	56.91	1.0561	1.0413	−1.40	+0.00	+1.10	+3.13
T05	R	62.32	61.04	9.69	9.68	55.18	53.93	1.0409	1.0421	+0.11	−0.01	−1.25	−2.05
T06	L	69.85	72.24	10.68	10.91	63.06	63.33	1.0557	1.0277	−2.65	+0.23	+0.27	+3.42
T07	R	76.03	75.83	9.77	9.91	67.43	67.11	1.0154	1.0157	+0.03	+0.14	−0.32	−0.26
T08	R	69.03	69.27	9.77	9.65	61.38	61.07	1.0307	1.0209	−0.95	−0.12	−0.31	+0.35
T09	R	62.34	62.38	7.71	7.51	56.54	55.69	1.0306	1.0131	−1.70	−0.20	−0.85	+0.06
T10	L	63.07	62.78	9.12	9.04	56.63	55.39	1.0425	1.0263	−1.55	−0.08	−1.24	−0.46

IE, intercondylar eminence; Ost, intersection of the osteotomy line with the cranial cortex; DA, center of the distal articular surface. A, straight IE → DA distance (tibial mechanical axis); B, straight IE→Ost distance (proximal segment); C, straight Ost → DA distance (distal segment); NEL = (B + C)/A. Δ% = 100 × (NEL_post − NEL_pre)/NEL_pre. ΔB and ΔC are absolute changes in millimeters (post − pre). ΔA (%) = 100 × (A_post − A_pre)/A_pre, reported as an internal check on the stability of the normalizing reference length between time points; pre, preoperative; post, immediately postoperative.

**Table 3:** GVI by time point and changes in GVI from the preoperative value (CTWO + TTT, 10 stifles; TPLO + TTT, 10 stifles).

ID	Limb	Pre GVI	Post GVI (Δ%)	Follow-Up GVI (Δ%)	Pattern	Technique	Follow-Up (Weeks)
C01	R	1.024	0.947 (−7.5%)	0.857 (−16.3%)	Sustained descent	CTWO + TTT	8–10
C02	L	0.816	0.638 (−21.8%)	0.609 (−25.4%)	Sustained descent	CTWO + TTT	8–10
C03	L	1.058	0.914 (−13.6%)	1.144 (+8.2%)	Descent, then recovery	CTWO + TTT	8–10
C04	R	1.176	0.946 (−19.6%)	0.877 (−25.4%)	Sustained descent	CTWO + TTT	8–10
C05	L	1.293	0.918 (−29.0%)	1.031 (−20.2%)	Descent, then partial recovery	CTWO + TTT	8–10
C06	R	0.937	0.801 (−14.5%)	0.934 (−0.4%)	Descent, then recovery	CTWO + TTT	8–10
C07	L	1.397	1.180 (−15.5%)	1.190 (−14.8%)	Descent, then maintenance	CTWO + TTT	8–10
C08	R	1.149	1.079 (−6.1%)	1.207 (+5.1%)	Descent, then recovery	CTWO + TTT	8–10
C09	R	1.455	1.273 (−12.5%)	1.252 (−14.0%)	Sustained descent	CTWO + TTT	8–10
C10	L	1.100	1.030 (−6.4%)	1.195 (+8.6%)	Descent, then recovery	CTWO + TTT	8–10
**Mean**		**1.141**	**0.973 (−14.6%)**	**1.030 (−9.5%)**			
T01	R	1.059	1.200 (+13.3%)	1.185 (+11.9%)	Elevation, then maintenance	TPLO + TTT	8–10
T02	R	1.314	1.215 (−7.6%)	1.273 (−3.2%)	Slight descent, then recovery	TPLO + TTT	8–10
T03	L	1.138	1.029 (−9.6%)	1.119 (−1.7%)	Descent, then recovery	TPLO + TTT	8–10
T04	R	1.113	0.995 (−10.6%)	1.203 (+8.0%)	Descent, then recovery	TPLO + TTT	8–10
T05	R	0.764	0.842 (+10.2%)	0.827 (+8.3%)	Elevation, then maintenance	TPLO + TTT	8–10
T06	L	1.045	1.066 (+2.0%)	0.990 (−5.3%)	Elevation, then slight descent	TPLO + TTT	8–10
T07	R	1.054	1.075 (+2.0%)	1.053 (−0.1%)	Position preserved	TPLO + TTT	8–10
T08	R	1.082	1.197 (+10.6%)	1.185 (+9.5%)	Elevation, then maintenance	TPLO + TTT	8–10
T09	R	1.036	1.012 (−2.3%)	1.064 (+2.7%)	Position preserved	TPLO + TTT	8–10
T10	L	0.873	0.953 (+9.2%)	0.909 (+4.2%)	Position preserved (elevation)	TPLO + TTT	8–10
**Mean**		**1.048**	**1.058 (+1.7%)**	**1.081 (+3.5%)**			

Bold rows indicate group means.

**Table 4:** Summary of the changes in the postoperative and follow-up patellar position (CTWO + TTT, 10 stifles; TPLO + TTT, 10 stifles).

Variable	CTWO + TTT (*n* = 10)	TPLO + TTT (*n* = 10)
Postoperative patellar descent (post GVI < pre)	10/10 (100%)	4/10 (40%)
Mean postoperative GVI Δ%	−14.6%	+1.7%
Mean follow-up GVI Δ% (vs. preoperative)	−9.5%	+3.5%
Sustained descent (further descent through follow-up)	4/10 (C01, C02, C04, C09)	0/10

**Table 5:** Overall comparison of the radiographic outcomes of the two procedures.

Variable	CTWO + TTT	TPLO + TTT	*p*-Value
NEL Δ% (mean ± SD)	−5.65 ± 1.29 (median −5.95; IQR −6.47, −5.10)	−0.98 ± 0.93 (median −1.17; IQR −1.53, −0.06)	<0.001 *
NEL Δ% variance	1.66	0.86	(CTWO 1.9× larger)
NEL Δ% range	−7.30 to −2.87	−2.65 to +0.11	—
Postoperative GVI Δ% (mean ± SD) †	−14.6 ± 7.3 (median −14.1; IQR −18.6, −8.8)	+1.7 ± 9.0 (median +2.0; IQR −6.2, +10.0)	0.002 ‡
Stifles with patellar descent †	10/10 (100%)	4/10 (40%)	—
Follow-up GVI Δ% (mean ± SD) †	−9.5 ± 13.5 (median −14.4; IQR −19.3, +3.7)	+3.5 ± 5.9 (median +3.4; IQR −1.3, +8.2)	—
BHS (mean ± SD)	2.70 ± 0.48 (median 3.0; IQR 2.25, 3.0)	3.90 ± 0.32 (median 4.0; IQR 4.0, 4.0)	<0.001 *
Complete union (BHS = 4)	0 (0%)	9 (90%)	<0.001 * (Fisher)

* Mann–Whitney U test or Fisher’s exact test. † GVI items are based on 20 stifles (CTWO 10, TPLO 10). ‡ Between-group postoperative GVI Δ% based on the Mann–Whitney U test, *p* = 0.002. NEL and BHS items are based on 20 stifles.

## Data Availability

The data presented in this study are openly available in Zenodo at https://doi.org/10.5281/zenodo.21457227 (reference number: zenodo.21457227).

## Abbreviations

The following abbreviations are used in this manuscript:

BHS	Bone Healing Score
CrCL	Cranial cruciate ligament
CTWO	Cranial tibial wedge osteotomy
DA	Center of the distal articular surface
ETL	Effective tibial length
GVI	Guénégo–Verwaerde index
IE	Intercondylar eminence
MA	Tibial mechanical axis
MPL	Medial patellar luxation
NEL	Normalized effective tibial length
Ost	Intersection of the osteotomy line with the cranial cortex
PJSL	Patellar joint-surface length
TPA	Tibial plateau angle
TPLO	Tibial plateau leveling osteotomy
TTT	Tibial tuberosity transposition
